# Serum Cholinesterase, C-reactive Protein, Interleukin 6, and Procalcitonin Levels as Predictors of Mortality in Patients in the Intensive Care Unit

**DOI:** 10.4274/TJAR.2023.231349

**Published:** 2023-10-24

**Authors:** Qin Liu, Xiaoguang Fan, Wenjuan Cui, Xincheng Wang, Zhaolong Zhang, Naizhi Wang, Lujun Qiao

**Affiliations:** 1 Intensive Care Unit, Shengli Oilfield Central Hospital, Dongying, China; 2 Department of Respiratory and Critical Care Medicine, Shengli Oilfield Central Hospital, Dongying, China; + The authors share first authorship

**Keywords:** Cholinesterase, C-reactive protein, intensive care, interleukin-6, procalcitonin

## Abstract

**Objective:**

The prognostic utility of inflammatory markers in survival has been suggested in patients with cancer; however, evidence on their prognostic value in severely ill patients is very limited. We aimed to explore the prognostic value of cholinesterase (ChE), C-reactive protein (CRP), interleukin-6 (IL-6), and procalcitonin (PCT) in predicting mortality in patients from the intensive care unit (ICU).

**Methods:**

Serum levels of ChE, CRP, IL-6 and PCT were measured in ICU patients from December 13^th^, 2019 to June 28^th^, 2022. We assessed the predictive power of ChE, CRP, IL-6, and PCT using the receiver operating characteristic (ROC) curves. Furthermore, we evaluated their diagnostic accuracy by comparing the areas under the ROC curve (AUCs) along with their corresponding 95% confidence intervals (CIs). The cut-off values were determined to dichotomise these biomarkers, which were then included in multivariable logistic regression models to examine their relationship with ICU mortality.

**Results:**

Among 253 ICU patients included in the study, 66 (26%) died during the ICU stay. The AUCs to predict ICU mortality were 0.643 (95% CI, 0.566-0.719), 0.648 (95% CI, 0.633-0.735), 0.643 (95% CI, 0.563-0.723) and 0.735 (95% CI, 0.664-0.807) for ChE, CRP, IL-6 and PCT, respectively. After adjusting for age, sex and disease severity, lower ChE level (<3.668 × 10^3^ U L^-1^) and higher levels of CRP (>10.546 mg dL^-1^), IL-6 (>986.245 pg mL^-1^) and PCT (>0.505 μg L^-1^) were associated with higher mortality risk, with odd ratios of 2.70 (95% CI, 1.32-5.54), 4.99 (95% CI, 2.41-10.38), 3.24 (95% CI, 1.54-6.78) and 3.67 (95% CI, 1.45-9.95), respectively.

**Conclusion:**

ChE, CRP, IL-6 and PCT were independent ICU mortality risk factors in severely ill patients. Elevated PCT levels exhibited better predictive value than the other three biomarkers that were evaluated.

**Main Points**

• Critically ill patients who did not survive in the intensive care unit exhibited elevated levels of C-reactive protein (CRP), interleukin-6 (IL-6), and procalcitonin (PCT), while their cholinesterase (ChE) levels were decreased.

• Serum PCT levels may have a better prognostic value compared to ChE, CRP and IL-6 in critically ill patients.

• Special attention should be given to the quality of care for critically ill patients at high risk of nosocomial infection.

## Introduction

Patients who are hospitalized in the intensive care unit (ICU) as a result of severe illness or injury face a greater risk of mortality when compared to other patients receiving inpatient care. One prospective, multi-centre study in China reported a hospital mortality rate of 20.3% in ICU settings.^1^ Patients in the ICU consume a large proportion of medical resources; therefore, identifying critically ill patients with higher likelihood of mortality is important for resource allocation as well as for improvement in quality of care in clinical practice. Nosocomial infections and severe sepsis are major contributors to mortality in patients in the ICU and provide better access points for interventions compared to other mortality risk factors such as advanced age, disease severity, and mechanical ventilation.^2^ Thus, identifying biomarkers of infection and sepsis are important for initiating appropriate treatments at an early stage and predicting prognosis.

The traditionally used markers of infection and sepsis include C-reactive protein (CRP), interleukin-6 (IL-6) and procalcitonin (PCT). CRP, the most commonly used marker of acute inflammation, is a valuable tool in monitoring treatment response.^3^ IL-6 is an early predictor that can indicate inflammation before the elevation of circulating CRP levels.^4^ In addition, PCT has been proposed as the most valuable marker of infection as it is a better indicator of the severity of systemic inflammatory response compared to other biomarkers.^5^ Although PCT, IL-6 and several other inflammatory markers have been regarded as prognostic indicators of survival in patients with cancer, evidence on their prognostic value in septic patients in the ICU is very limited and inconsistent*.*^6,7^

In addition to the classic inflammatory biomarkers, several novel markers such as cholinesterase (ChE) have been considered as predictive factors for mortality. Serum ChE is primarily synthesised in hepatocytes before its release into peripheral blood.^8^ ChE activity is reduced in severe clinical conditions such as liver dysfunction, malnutrition, heart disease, sepsis, inflammation, stress and cancer.^8,9^ The serum level of ChE has been reported to inversely correlate with levels of inflammatory biomarkers such as IL-6 and tumour necrosis factor α.^10^ Furthermore, ChE has been demonstrated to be related with mortality and complications in patients with stroke,^11^ cancer^12^ or in those undergoing surgery.^13^ Therefore, serum ChE level is often used as a routine biochemical parameter in clinical diagnostic procedures. However, no study to date has examined changes in serum ChE level in ICU patients and its prognostic value for mortality remains unclear.

In the present study involving patients admitted to the ICU, we measured serum ChE levels and examined its correlation with CRP, IL-6 and PCT. We also compared the prognostic value of these biomarkers in predicting mortality among ICU patients.

## Methods

### Study Population

From December 13^th^, 2019 to June 28^th^, 2022, we conducted a cohort study in the ICU of Shengli Oilfield Central Hospital, Shangdong, China. A total of 299 ICU patients were randomly selected. Patients who were under 18 years of age; those who were immunocompromised due to malignancy, human immunodeficiency virus infection or other reasons; those who were pregnant; and those who were without legal representatives or were unable to provide written informed consent were excluded. The final study cohort included 253 patients, who were followed from the date of ICU admission until death due to any cause or until ICU discharge. The study was approved by the Ethics Committee of Shengli Oilfield Central Hospital (no. Q/ZXYY-ZY-YWB-LL202149).

### Sample Collection and Laboratory Tests

After ICU admission, a 5 mL peripheral venous blood sample was collected from each patient via venipuncture. The collected samples were then centrifuged at 3500 r min for 5 min, and the resulting serum samples were stored at a temperature of -80 °C for subsequent analyses. To determine serum CRP levels, we used a commercially available CRP test kit (Mindray, Shenzhen, China) based on the immunoturbidimetric method. IL-6 and PCT levels were measured by electrochemiluminescence using the Elecsys IL-6 and BRAHMS PCT (Roche) kits, respectively. Serum ChE levels were determined using a kit from Shanghai Gao Chuang Medical Technology, Shanghai, China based on the butyrylthiocholine method.

### Data Collection

The following data were collected: dates of ICU admission and discharge, age, sex, Acute Physiology and Chronic Health Evaluation (APACHE) II score and underlying indications for ICU admission. The outcome of interest was all-cause ICU mortality.

### Statistical Analysis

Comparisons of continuous variables between the survival and non-survival groups were performed using the Mann-Whitney U or Student’s t-test, and comparisons of categorical variables between the two groups were performed using the χ*^2^* test or Fisher’s exact test. Correlations of ChE with CRP, IL-6, and PCT was evaluated by Spearman’s nonparametric correlation coefficient (rho).

The ability of CRP, IL-6, PCT and ChE levels to predict mortality was measured by using receiver operation characteristic curves (ROCs). The cutoff values were determined by maximum area under the ROC curve (AUC), and sensitivity and specificity were calculated for each biomarker. ROC comparisons were conducted using a nonparametric method.

Cutoff values were used to dichotomise CRP, IL-6, PCT and ChE, which were then included in the logistic regression model to examine their relationship with all-cause ICU mortality. The multivariable logistic regression model was adjusted for patient age, sex and disease severity determined by APACHE II score. To investigate whether the relationship of inflammatory markers with ICU mortality differed by sex, the study patients were stratified by sex and its interaction with biomarkers was analysed by including the interaction term.

All statistical tests were two-sided with an α-level of 0.05. All analyses were performed using SAS version 9.4 (SAS Institute, Cary, NC, USA) and R version 5.3.0.

## Results

### Overall Cohort Characteristics

In the present study, 66 of the 253 patients (26%) did not survive. Compared to the survivors, the non-survivors were more likely to be older males and to have higher APACHE II scores (Table 1). Median serum ChE level was lower in the non-survivors than in the survivors [2.93 (2.23-3.83) × 10^3^ U L^-1^ vs. 3.87 (2.80-5.41) × 10^3^ U L^-1^). In contrast, compared to the survivors, the non-survivors had significantly higher serum levels of CRP [11.48 (6.87-16.99) vs. 8.24 (4.37-10.72) mg dL^-1^], IL-6 [815.86 (100.01-3916.66) vs. 190.67 (44.68-799.97) pg mL^-1^] and PCT [0.81 (0.53-1.93) vs. 0.25 (0.07-0.72) µg L^-1^]. Serum ChE level was significantly correlated with CRP (rho, -0.22; *P* < 0.001), IL-6 (rho, -0.16; *P*=0.009) and PCT (rho, -0.15; *P*=0.01).

### ROC Analysis

**Figure 1:**
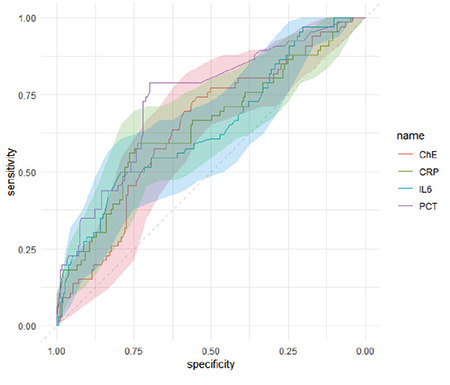
Receiver operating characteristic curve analysis for predicting mortality in the intensive care unit according to serum levels of cholinesterase, interleukin-6, C-reactive protein and procalcitonin. ChE, cholinesterase; IL-6, interleukin-6; CRP, C-reactive protein; PCT, procalcitonin.

In analyses to evaluate the ability of inflammatory biomarkers in predicting ICU mortality revealed that the AUCs of ChE, CRP, IL-6 and PCT were 0.643 [95% confidence interval (CI), 0.566-0.719], 0.648 (95% CI, 0.633-0.735), 0.643 (95% CI, 0.563-0.723) and 0.735 (95% CI, 0.664-0.807), respectively (Figure 1). The optimal cut-off values of ChE, CRP, IL-6 and PCT for ICU mortality were 3.668 × 10^3^ U L^-1^ (sensitivity, 74.2%; specificity, 55.6%), 10.546 mg dL^-1^ (sensitivity, 59.1%; specificity, 73.8%), 986.245 pg mL^-1^ (sensitivity, 50.0%; specificity, 79.1%) and 0.505 µg L^-1^ (sensitivity, 78.8%; specificity, 70.1%), respectively. Comparison of the ROC curves revealed no significant difference in the clinical values of ChE and CRP (*P*=0.93) or those of cholinesterase and IL-6 (*P*=0.99), although the clinical values of ChE and PCT exhibited a marginally significant difference (*P*=0.06).

### Relationship Between Inflammatory Markers and Mortality

In logistic regression including the dichotomised values of study biomarkers according to the optimal cutoff values revealed that patients with low ChE levels (≤3.668 × 10^3^ U L^-1^) were at higher risk of ICU mortality than these with high ChE levels [adjusted odds ratio (OR), 2.70; 95% CI, 1.32-5.54] (Table 2). Additionally, patients with higher serum levels of CRP (>10.546 mg dL^-1^), IL-6 (>986.245 pg mL^-1^) or PCT (>0.505 µg L^-1^) were at higher risk of ICU mortality than those with low levels of these biomarkers, with adjusted ORs of 4.99 (2.41-10.38), 3.24 (1.54-6.78) and 3.67 (1.45-9.95), respectively.

### Analysis of ICU Mortality Stratified by Sex

Cholinesterase, IL-6 and PCT did not significantly interact with patient sex for ICU mortality risk (*P*=0.94, *P*=0.50 and *P*=0.25, respectively), whereas CRP did (*P*=0.04) (Table 3). The adjusted ORs for ChE and CRP were higher in female patients than in male patients, although the 95% CIs were wider due to limited number of women. Moreover, the relationship between IL-6 and ICU mortality risk was slightly stronger in male patients than in female patients.

## Discussion

In the present study evaluating critically ill patients in the ICU, the mortality rate was 26%, which was comparable to that reported in other countries (17-38%).^1,14^ Nosocomial infection is a major contributor of ICU mortality. Therefore, special attention should be given to the quality of care for patients who are at high risk of nosocomial infection. The utilization of inflammatory biomarkers in screening procedures can aid in evaluating the risk of mortality among ICU patients.

In the present study, we explored the clinical diagnostic value of CRP, IL-6 and PCT in ICU patients, as well as that of ChE as a potential novel biomarker. In our findings, we observed higher serum levels of CRP, IL-6, and PCT, along with lower levels of ChE, in non-surviving patients when compared to the survivors. The ROC curve analyses revealed that the AUC was greatest for PCT. Patients with ChE, CRP, IL-6 or PCT levels of <3.67 × 10^3^ U L^-1^, >10.55 mg dL^-1^, >986.25 pg mL^-1^ or >0.51 µg L^-1^, respectively, were at increased risk of ICU mortality even after taking control of patients’ age, sex and the APACHE II score. In addition, the risk magnitudes for ChE and CRP were stronger in female patients than in male patients.

In patients with liver dysfunction, the activity of ChE, a crucial enzyme, is decreased due to reduced synthesis, in contrast to other serum enzymes whose activity levels are elevated due to cell membrane damage and the subsequent cellular release.^15^ Additionally, decreased ChE activity was recently reported to be closely associated with malnutrition, cancer and severe inflammation.^9,12,13^ However, the value of ChE activity as a diagnostic tool for mortality prognosis is often overlooked in ICU settings. To our best of knowledge, the present study is the first to investigate the association between serum ChE levels and ICU mortality risk. Our results demonstrating the significant and inverse correlation of ChE with the other three inflammatory biomarkers, i.e. CRP, IL-6 and PCT, are in line with the findings of a previous study.^10^ Surprisingly, serum ChE levels were not decreased in the non-survivor patients in the present study. The ROC curve analysis indicated that the sensitivity and specificity of ChE at a cut-off value of <3.67 × 10^3^ U L^-1^ were not high enough for its utility as a diagnostic marker in the ICU. Nevertheless, ChE, as a dichotomised variable, was associated with increased ICU mortality risk regardless of age, sex or disease severity. Therefore, ChE might be considered as a potential tool to assist clinicians in the prognostic evaluation of patients in the ICU.

CRP, IL-6 and PCT are well-known inflammatory markers; however, evidence on their potential as predictors of ICU mortality is limited. In one study including four ICUs in China, the serum level of CRP, but not PCT, was associated with mortality.^6^ Furthermore, the inclusion of serum CRP as a prognostic predictor improved risk reclassification. The cut-off CRP and PCT values used in that study were slightly lower than those found in the present study, which might be due to the higher rate of infection in the current study cohort. Another study in Germany reported that IL-6, rather than PCT or CRP, might potentially serve as a reliable predictor of mortality during the early stage of ICU patients with the early onset of fever;^4^ however, the authors also stated that the prognostic value of PCT might be better than that of IL-6 in patients with established SIRS and sepsis. In the present study, the sensitivity and specificity of PCT in predicting ICU mortality were 78.8% and 70.1% at a cutoff value of 0.5 µg L^-1^, which was comparable to that reported by the study in Germany. Therefore, our findings suggest that PCT has better prognostic value than ChE, CRP and IL-6 in critically ill patients. Nevertheless, our analyses also suggest serum CRP, IL-6 and PCT as plausible risk factors based on their association with increased ICU mortality risk after controlling for potential confounders.

### Study Limitations

This study has several limitations to be noted. First, this was a single-centre study with a small sample size and lacked power to evaluate the predictive value of these four biomarkers when used in combination. Second, information on some important confounders, such as socio-economic status, body mass index, preexisting clinical conditions and relevant treatments, was not available for analyses in the present study.

## Conclusion

Altered serum levels of ChE, CRP, IL-6 and PCT were associated with increased risk of ICU mortality in severely ill patients. Elevated PCT level may be useful as a prognostic marker in these patients. Further studies with larger cohorts are warranted to improve the predictive values of these four biomarkers used in combination.

## Figures and Tables

**Table 1:**
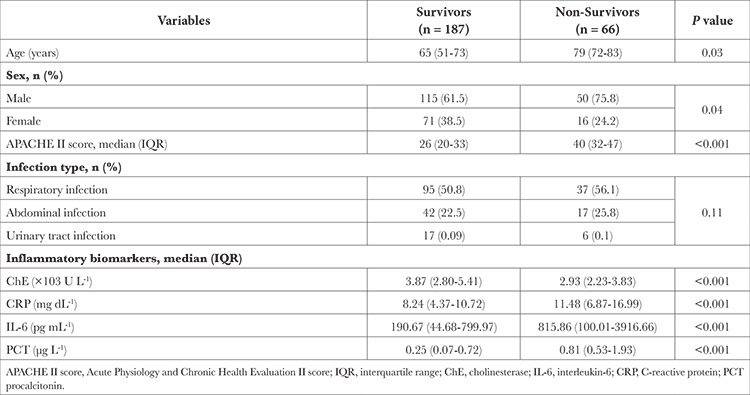
Characteristics of Survivors and Non-Survivors in the Intensive Care Unit

**Table 2:**
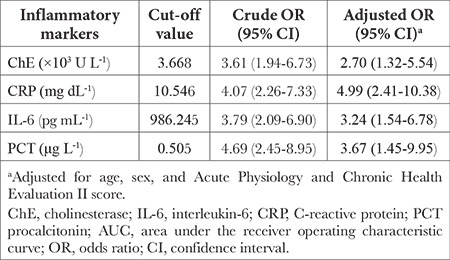
Relationship of ChE, CRP, IL-6 and PCT With Mortality in Patients in the Intensive Care Unit

**Table 3:**
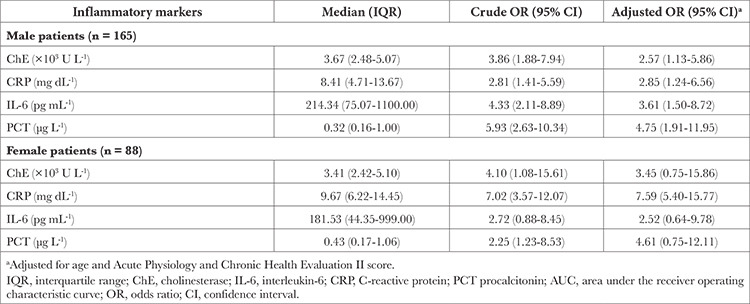
Relationship of ChE, CRP, IL-6 and PCT with Mortality in the Intensive Care Unit in Patients Stratified by Sex
